# INADEQUATE SLEEP ASSOCIATED WITH FRAILTY DEVELOPMENT AND FALL RISK IN LOW-INCOME OLDER ADULTS

**DOI:** 10.1093/geroni/igae098.4006

**Published:** 2024-12-31

**Authors:** Abigail Tice, Chen Chen, Varadraj Gurupur, Boon Peng Ng, David Fukuda, Janet Lopez, Hwan Choi, Ladda Thiamwong

**Affiliations:** University of Central Florida, Orlando, Florida, United States; University of Central Florida, Orlando, Florida, United States; University of Central Florida, Orlando, Florida, United States; University of Central Florida, Orlando, Florida, United States; University of Central Florida, Orlando, Florida, United States; University of Central Florida, Orlando, Florida, United States; University of Central Florida, Orlando, Florida, United States; University of Central Florida, Orlando, Florida, United States

## Abstract

Sleep, which is important for improving brain performance and overall health, declines with aging, and older adults from disadvantaged socioeconomic groups are more likely to have poorer sleep. Frailty, the decline of physiological systems, is associated with poor sleep parameters (e.g., less deep sleep and sleep duration) in older adults. As frailty progresses, fall risk and fear of falling (FOF) increase, increasing the likelihood of a fall that can result in serious injury, hospitalization, and even death. The relationship between sleep duration and frailty, fall risk, and FOF is not well understood, therefore, the objective of this study was to investigate the relationships between them. The Fatigue, Resistance, Ambulation, Illness, and Loss of weight (FRAIL), Stopping Elderly Accidents, Death, and Injuries (STEADI), short Fall-Efficacy International (FES-I), and Pittsburgh Sleep Quality Index (PSQI) questionnaires were administered to 124 community-dwelling, low-income older adults aged 60 years or more in Orlando, Florida. Spearman correlation analysis revealed associations between sleep duration and FRAIL (p=0.0258, r=-0.1741), STEADI (p=0.0010, r=-0.2537), and short FES-I (p=0.0083, r=-0.2056). While unpaired t-test analysis revealed no difference in short FES-I between low sleep (< 7 hours; n=83) and healthy sleep (7-8 hours; n=72), FRAIL (p=0.0248) and STEADI scores (p=0.0338) significantly increased with low sleep duration. In conclusion, inadequate sleep duration appears to increase the development of frailty and fall risk in community-dwelling older adults. Screening older adults for sleep problems and interventions to improve sleep duration have the potential to reduce frailty and fall risk in low-income older adults.

